# DTTF-Sim: A Digital Twin-Based Simulation System for Continuous Autonomous Driving Testing

**DOI:** 10.3390/s25113447

**Published:** 2025-05-30

**Authors:** Zhigang Liang, Jian Wang, Tingyu Zhang, Xinyu Yong

**Affiliations:** The College of Computer Science and Technology, Jilin University, No. 2699 Qianjin Street, Changchun 130000, China; liangzg22@mails.jlu.edu.cn (Z.L.); zhangty21@mails.jlu.edu.cn (T.Z.); yongxy22@mails.jlu.edu.cn (X.Y.)

**Keywords:** autonomous driving testing, traffic simulation, digital twins

## Abstract

As autonomous driving technology matures, the focus shifts to enhancing the safety and reliability of these systems. Simulation testing is a critical method for efficiently and rapidly validating the performance of autonomous vehicles (AVs). A robust AV system requires extensive testing across a wide range of scenarios and iterative improvements. However, current simulation systems have limitations in supporting diverse scenarios, often relying on expert-designed situations. To address these challenges, we introduce DTTF-Sim, a novel simulation system based on Digital Twin technology for traffic flow. DTTF-Sim aims to accurately replicate real-world traffic flow conditions, offering continuous long-term simulation capabilities for AV testing. The system can simulate detailed dynamic traffic scenarios with a focus on interactions between multiple vehicles and between AVs and background traffic vehicles, modeling the strategic decision-making processes that occur in these encounters. This paper outlines the architecture and functionalities of DTTF-Sim, highlighting its ability to overcome the shortcomings of existing simulation platforms. We demonstrate the effectiveness of DTTF-Sim through case studies and experimental results, showing its potential to significantly advance the development and testing of autonomous driving technologies.

## 1. Introduction

Autonomous driving technology is reshaping our modes of transportation, enhancing both the convenience and safety of travel. However, to achieve this transformation, it is critical that the internal algorithms of Autonomous Driving Systems (ADSs) are capable of safely and reliably handling the myriad dynamic scenarios encountered in the real world. Therefore, before autonomous driving technology can be deployed, it must undergo comprehensive and rigorous safety validation. Currently, the testing of autonomous driving primarily relies on two methods: virtual simulation testing and physical road testing. According to a study by RAND Corporation [1], to ensure the reliability of an ADS, it should complete at least 150 million miles of driving tests prior to market release. Virtual simulation testing, due to its cost-effectiveness and high efficiency, is considered an ideal approach for validating the performance of ADS.

Current simulation tools can be broadly classified into four categories: flow-based simulators, scenario-based simulators, vehicle-based simulators, and data-driven simulators. Flow-based simulators, such as SUMO [2], PTV Vissim [3], Aimsun [4], and Paramics [5], can effectively simulate vehicle movements within road networks to evaluate the effectiveness of various traffic management and planning strategies. However, their simplified approach to vehicle simulation limits their ability to accurately represent the attributes of autonomous vehicles, particularly their kinematic features [2]. Scenario-based simulators, such as Virtual Test Drive (VTD) [6] and CARLA [7], use standard scenario files (e.g., OpenSCENARIO) to depict interactions between vehicles and provide kinematic attributes as well as basic dynamic system features, such as engine response and braking performance. Nevertheless, these scenario files require predefined relative positions, velocity changes, and interaction rules between vehicles, driven by behavior trees [8]. This dependency restricts the simulators’ ability to comprehensively evaluate complex game-theoretic behaviors among vehicles in dynamic environments. Vehicle-based simulators, such as Matlab Simulink (version R2023a) and CarSim (version 2023.1), focus on accurately modeling vehicle dynamics. These tools analyze and assess the impacts of individual vehicle components, such as tires, suspension systems, and engines, on overall vehicle performance. Data-driven simulators rely on deep neural network models and high-quality road datasets, such as SimNet [9] and TrafficGen [10]. These simulators leverage neural network models to extract vehicle trajectory features from the datasets, thereby enabling the regeneration of original traffic scenarios. However, current data-driven simulators are limited to providing fragmented test scenarios and have not yet achieved the capability to support long-duration continuous testing.

Our research aims to provide an authentic, comprehensive, diverse, and interactive traffic flow environment for ADS, thereby enhancing their performance in complex and dynamic scenarios. To this end, we developed the Digital Twin Traffic Flow Simulator (DTTF-Sim), a novel platform grounded in digital twin technology. DTTF-Sim is capable of rapidly acquiring and processing high-fidelity data from actual traffic conditions. DTTF-Sim applies the principles of digital twinning to recreate traffic flow patterns within a virtual simulation environment. Moreover, to augment the realism and interactivity of the simulation, DTTF-Sim is integrated with the CARLA simulation platform for co-simulation.

Compared to existing simulation platforms, DTTF-Sim offers the following significant advantages:

1. DTTF-Sim can faithfully reproduce traffic data in the simulation environment, providing accurate vehicle dynamics information. This ensures a realistic and reliable testing environment for autonomous driving systems.
2. DTTF-Sim supports participation in the entire process by autonomous driving systems, from sensor data input to control command output. It allows these systems to engage in complex interaction and strategic decision-making within the simulated traffic flow, thereby evaluating their decision-making and response capabilities in dynamic scenarios.
3. DTTF-Sim provides a long-term continuous simulation environment, enabling comprehensive assessment of system stability and reliability over extended periods. Unlike other simulators that may be limited to short or fragmented test scenarios, DTTF-Sim supports sustained testing under various complex traffic conditions.

The remainder of this paper is organized as follows. Section 2 reviews related work on existing simulation platforms and their limitations. Section 3 formally defines the problem and presents the concept of digital twin mapping for traffic flow. Section 4 provides an overview of the DTTF-Sim framework, followed by a detailed description of each core module in Section 5. Section 6 presents the implementation details and evaluates the system’s performance through simulation experiments. Finally, Section 7 concludes the paper and outlines future research directions.

## 2. Related Work

This section aims to provide a concise overview of the application and key contributions of existing virtual simulation tools in autonomous driving system testing.

### 2.1. Flow-Based Simulators

Traffic flow simulation tools not only focus on the overall behavior of traffic flow but also depict individual vehicle behaviors such as car-following and lane-changing at a microscopic level. At a macroscopic level, these simulators describe the collective movement of vehicles on the road using continuous flow models or discrete flow models, enabling efficient handling of large-scale traffic networks and rapid evaluation of traffic management and control strategies. PTV Vissim (version 2023). is a widely used microscopic traffic simulation software in the fields of traffic engineering and urban planning. It can simulate individual vehicle behaviors within road networks and evaluate the performance of transportation infrastructure and measures. Through the COM interface provided by the Vissim platform, it is possible to analyze and assess the impact of AI-based products on traffic performance [11,12,13,14]. Moreover, the platform supports microscopic traffic simulations involving autonomous driving systems, allowing for the evaluation of automated transportation in terms of efficiency, safety, equity, and environmental impacts [15,16]. By integrating the Aimsun traffic simulation platform with the Robot Operating System (ROS), ref. [17] provides an effective solution for evaluating the performance of connected and automated vehicles in traffic networks. Paramics can simulate the intentions, decisions, and subsequent actions of each driver as they move through the road network [18,19]. Simulation of Urban Mobility (SUMO) (version 1.18.0). is an open-source microscopic traffic flow simulation software widely used for modeling and analyzing traffic systems. SUMO not only provides an intuitive graphical user interface but also features a powerful programming interface (Libsumo), enabling researchers to flexibly customize and control complex traffic scenarios. These capabilities allow for in-depth investigation of the dynamic features of traffic flow and optimization of autonomous driving system performance. Consequently, SUMO has become an essential research tool in the fields of traffic engineering and autonomous driving [20,21,22,23,24]. Flow-based simulation platforms are important tools for analyzing the impact of different traffic participants on traffic performance. However, for autonomous driving, these simulators typically simplify vehicle modeling to a point mass model, where each vehicle is represented by a single geometric point indicating its position. This simplification ignores the actual physical dimensions and shape of the vehicles, as well as their steering kinematic features, which are crucial for autonomous driving systems. These ignored properties are essential for accurately assessing the behavior and performance of autonomous vehicles.

Compared to existing simulation platforms, DTTF-Sim achieves a relatively balanced design in terms of simulation fidelity, interactivity, and scalability. Flow-based simulators, such as SUMO and VISSIM, are highly practical for large-scale traffic modeling and traffic management strategy evaluation. However, these tools typically model vehicles as point-mass objects, which limits their ability to accurately capture the essential kinematic and dynamic characteristics required for autonomous driving systems. In contrast, DTTF-Sim preserves vehicle dimensions and fundamental dynamic constraints, enabling more realistic modeling of interaction behaviors that closely resemble real-world traffic scenarios.

### 2.2. Scenario-Based Simulators

Scenario-based simulation platforms utilize rendering engines (such as OpenGL, Unreal, and Unity) to generate the simulated environment. These platforms describe controllable objects within the simulation using scenario files and employ behavior trees to control the movement of background objects [8]. Experienced scenario designers choreograph the actions of background objects. These background objects follow the predefined scenarios to interact with the test vehicle, thereby facilitating the completion of the testing tasks. Virtual Test Drive (VTD) is a comprehensive virtual traffic environment simulation platform developed by the German company Vires. It is specifically designed for creating, simulating, and evaluating complex traffic scenarios. VTD is a simulation tool that supports multiple standard traffic environment description formats, including OpenDRIVE, OpenSCENARIO, and OpenCRG. As a result, VTD has become an essential tool for researchers in the development of autonomous driving and Advanced Driver Assistance Systems (ADAS). Researchers utilize VTD for various autonomous driving-related studies, including sensor simulation [25,26] and automatic generation of test scenarios [27]. The CARLA simulation platform was jointly developed by the Computer Vision Center in Barcelona, Spain, and Intel Labs in 2017. It is an open-source simulator specifically designed for autonomous driving research. CARLA offers flexible information exchange interfaces, enabling efficient communication with leading autonomous driving systems such as Autoware [28] and Apollo [29], thus providing a highly efficient and adaptable platform for autonomous driving research. Based on the CARLA platform, researchers can perform autonomous driving algorithm optimization, performance evaluation, and the generation and simulation of complex traffic scenarios, thereby advancing the development and testing of autonomous driving technologies [30,31,32,33]. To address the inadequacies of existing simulation systems in supporting a diverse range of scenarios, [34] introduces the Long-term Interactive Multi-scenario Traffic Simulator (LimSim), which is designed to provide long-term continuous simulation capabilities within an urban road network. Other scene-based simulators include PreScan (version 2023.1), AirSim (version: v1.8.1) [35], NVIDIA Drive Constellation, LGSVL (version: 2021.3), PanoSim (version: V33) [36] and others.

Scenario-driven simulators, such as CARLA and VTD, rely on predefined scenario scripts and behavior trees to control traffic participants in the environment. This design inherently constrains the diversity and spontaneity of interactions. By incorporating real-world traffic data and leveraging digital twin modeling techniques, DTTF-Sim reconstructs traffic behaviors with greater realism and diversity without the need for manually defined scripts, thereby enhancing the expressiveness and variability of simulated scenarios.

### 2.3. Vehicle Based Simulator

Vehicle-based simulators are software tools designed to simulate and analyze the dynamic behavior of vehicles. By employing mathematical models, these simulators predict vehicle motion and performance under various driving conditions, assisting engineers in avoiding costly physical experiments. These tools not only simulate dynamic features such as acceleration, steering, and braking but also analyze the responses of suspension and steering systems. Additionally, they evaluate the operational states of powertrain components, such as engines and transmissions. Commonly used simulators include CarSim, CarMaker, Simulink, dSPACE and so on. Researchers utilized a co-simulation platform integrating CarSim and Simulink to validate path planning algorithms and path-following control strategies [37,38,39] for autonomous driving systems. Meanwhile, the CarMaker simulation platform was utilized for the systematic validation and evaluation of key functionalities, including sensor fault detection algorithms, trajectory planning, and trajectory tracking control. Furthermore, the dSPACE platform was used to comprehensively validate and evaluate the performance of decision-making systems [40] and collision avoidance algorithms [41]. Vehicle-based simulators are primarily used to validate whether control models can accurately simulate the dynamic behavior of real vehicles, while parameter calibration is employed to enhance the precision and adaptability of the models. However, existing simulators exhibit limitations in scenarios involving multi-vehicle interactions and game-theoretic decision-making. To address these challenges, integration with other types of simulators is required to comprehensively simulate collaborative decision-making in complex traffic environments.

Vehicle-based simulators (e.g., CarSim and Simulink) offer high accuracy in modeling vehicle dynamics and validating control strategies, but their capabilities are relatively limited when it comes to simulating multi-vehicle interactions and complex traffic environments. DTTF-Sim complements these simulators by introducing interaction modeling at the strategic level, thereby strengthening its utility in evaluating decision-making systems within autonomous driving frameworks.

### 2.4. Data-Driven Simulators

Traffic data contain a wealth of human driving experience and behavioral patterns, providing a crucial foundation for researchers to construct more complex and realistic traffic scenarios. Recent research trends have increasingly shifted towards mining behavioral features from large-scale traffic datasets to simulate more diverse and dynamic traffic environments. During simulation, autonomous driving systems—particularly the ego vehicle—can learn and emulate various driving behaviors and decision-making strategies from such data, enabling more accurate reproduction of driving responses under real-world road conditions.

At present, some research teams have collected real-world driving data based on high-definition map information to train and validate models for vehicle trajectory prediction, object detection, and other tasks. Representative efforts include nuScenes, nuPlan [42], and Waymo [43]. In addition, both nuPlan and Waymo provide user-friendly offline simulation platforms for researchers, where traffic scenarios are driven by either rule-based or machine learning-based agent models. These platforms have significantly alleviated the data scarcity bottleneck in autonomous driving training and have accelerated the development of the field. In these platforms, open-loop simulation is typically implemented through log replay, where background vehicles follow pre-defined trajectories strictly. In contrast, closed-loop simulation enables more dynamic interactions by employing rule-based or learning-based behavior models, allowing vehicles in the simulation to deviate from recorded trajectories and respond adaptively to the evolving environment.

In recent years, a growing number of studies have explored simulation frameworks that leverage deep learning techniques to automatically learn driving behaviors from large-scale traffic data and generate diverse traffic scenes. For example, SimNet [9] was the first to introduce a machine learning-based framework for traffic scene generation, learning directly from historical observational data to capture complex human driving behaviors and enhance the realism of the simulation. TrafficGen [10] is another representative learning-driven simulator, which employs an encoder–decoder neural network architecture to synthesize complete traffic scenes. In addition to generating realistic traffic flow, it can augment existing scenarios by adding new vehicles or extending fragmented trajectories. This flexibility enables TrafficGen to effectively enhance both the diversity and fidelity of generated traffic environments.

In summary, although data-driven simulators excel at reproducing historical traffic scenarios, their particle-based models for simulating real vehicle kinematics still face significant challenges in capturing realistic vehicle interactions. These limitations have motivated the research community to explore simulation systems with enhanced behavior generation capabilities and adaptive interaction mechanisms, aiming to support more comprehensive testing and validation of autonomous driving systems in the future.

In addition, compared to typical data-driven simulators such as nuPlan, Waymo-SimAgent, and SimNet, DTTF-Sim supports continuous and long-term real-world traffic simulation as well as sensor simulation, making it particularly suitable for comprehensive evaluation of system stability and robustness over extended periods. It should be noted that the performance of DTTF-Sim depends to some extent on the availability and spatial coverage of high-fidelity real-world traffic data. Moreover, the current modeling focus of DTTF-Sim lies in capturing macroscopic interactions among traffic participants and does not yet include fine-grained vehicle-level mechanical response modeling. Therefore, DTTF-Sim is especially suited for system-level verification and integrated evaluation of autonomous driving systems under complex and highly interactive traffic environments, serving as an efficient and general-purpose simulation platform. Table 1 provides a comparative and critical analysis of the advantages and limitations of four mainstream types of simulation platforms and the proposed DTTF-Sim system. Table 2 presents a systematic comparison between DTTF-Sim and other simulation platforms.

## 3. Problem Description

The Digital Twin (DT) is a high-fidelity virtual representation built using digital technologies which accurately maps and simulates physical entities in the real world. By integrating advanced sensing technologies, big data analysis, and sophisticated simulation models, the DT enables comprehensive modeling and real-time simulation of physical systems, thereby supporting performance prediction and optimization [44]. The problem of autonomous driving dynamic traffic flow based on digital twins aims to establish a twin mapping ($F_{twin}$) between real traffic flows (${\mathrm{TF}}_{r}$) and simulation (${\mathrm{TF}}_{s}$) environments, $F_{twin}:{\mathrm{TF}}_{r}\to {\mathrm{TF}}_{s}$. In this process, real traffic flow data (including near-real-time or historical data) are transformed into corresponding traffic flow in the virtual environment, ensuring that, within a given time interval, the virtual traffic flow remains consistent with the real traffic flow in terms of key features. Specifically, the twin mapping should satisfy the following conditions:

- the $F_{twin}$ should ensure the similarity between virtual and real traffic flow in several aspects, including road network, vehicle incoming, speed distribution, gap distribution, time headway distribution, lane density, lane-changing similarity, lane-center deviation and so on;
- the $F_{twin}$ should accurately replicate the kinematic features of both background vehicles and autonomous vehicles, ensuring that the vehicles in the simulation exhibit dynamic behaviors that align with those in the real world, such as a complete lane-changing process;
- the $F_{twin}$ should incorporate autonomous interaction capabilities between background vehicles and autonomous vehicles, as well as among background vehicles themselves, covering behaviors such as car-following, lane changing, and collision avoidance, thereby ensuring the overall consistency and dynamic evolution of traffic flow in the simulation environment.

It is important to note that this paper focuses exclusively on the twin mapping of traffic flow and does not address real-world environmental factors (e.g., weather, road quality). It is assumed that current mainstream scenario-based simulators can handle these aspects.

## 4. Framework Overview

**Figure 1:**
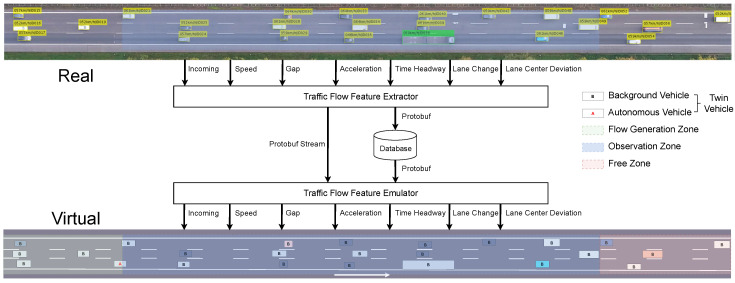
The overall architecture of DTTF-Sim, which is implemented on top of the CARLA simulator. It consists of three modules: the Traffic-Flow Feature Extractor Module, the Traffic-Flow Feature Emulator Module, and the Twin-Vehicle Module Real-world traffic features are extracted and used to guide the dynamic simulation of background and autonomous vehicles.

DTTF-Sim is a digital twin-based simulation platform built on top of the CARLA simulator. It is designed to accelerate the testing process of autonomous driving systems while significantly reducing the high costs associated with physical vehicle testing. To achieve this, DTTF-Sim captures real-world traffic flow features at fixed time intervals *T*. Subsequently, DTTF-Sim employs a traffic flow feature twinning module to replicate these features within the simulation environment, ensuring that the background vehicles in the twin system exhibit dynamic behaviors closely resembling those in the real-world traffic flow. The framework of DTTF-Sim is shown in Figure 1. DTTF-Sim consists of three core modules: traffic flow feature extractor module, traffic flow feature emulator module, and the twin-vehicle module.

The Traffic Flow Feature Extractor module is responsible for analyzing real-world vehicle trajectories, which consist of time-ordered points including timestamp, position, velocity, acceleration, and lane information. These raw trajectories, collected from high-resolution traffic datasets, serve as the input to the extractor. The module processes these data and outputs a structured set of traffic flow features, which include (1) Incoming Features (e.g., vehicle entry count, speed, size, and lateral offset), (2) Longitudinal Features (e.g., speed statistics, gaps, time headways, acceleration profiles), and (3) Lateral Features (e.g., lane center deviation and lane-changing behavior attributes). These features are extracted in fixed time intervals and stored using efficient protocols (e.g., Protobuf), enabling both stream-based and database-based retrieval.

The traffic flow feature emulator receives the extracted traffic features and reconstructs corresponding traffic flow dynamics within a virtual simulation environment. It generates and controls the behavior of background vehicles, ensuring that their interactions—such as car-following, lane-changing, and collision avoidance—accurately reflect the statistical and behavioral properties of the real-world data. Unlike traditional rule-based simulators, DTTF-Sim uses these feature-driven strategies to reproduce both macroscopic and microscopic traffic behaviors without relying on predefined scripts or behavior trees. This data-centric design enables DTTF-Sim to support long-duration continuous simulation and accurately emulate dynamic vehicle interactions. Notably, the emulator organizes the road environment into three zones: a flow generation zone, an observation zone, and a free zone, each serving a distinct role in the lifecycle of simulated vehicles.

- Flow Generation Zone: This is the entry region of the simulation where new background vehicles are dynamically created based on the extracted incoming traffic features, such as entry speed, size, and lateral offset. The emulator initializes vehicle states and assigns their paths according to lane-specific traffic flow parameters, ensuring alignment with real-world inflow conditions.
- Observation Zone: Positioned at the center of the simulation environment, this zone serves as the primary area for evaluating traffic interactions. Within this region, background vehicles execute car-following, lane-changing, and collision avoidance behaviors under the guidance of the feature emulator. The observation zone is designed to preserve fidelity to real-world traffic distributions and is typically the target area for testing autonomous vehicle performance and interaction.
- Free Zone: This terminal region marks the exit of vehicles from the simulation. Once vehicles cross into the free zone, they are safely destroyed by the emulator to release computational resources. This mechanism ensures efficient simulation management while preserving behavioral continuity up to the point of vehicle removal.

The twin vehicle system consists of two types of agents: background vehicles and autonomous vehicles. Background vehicles are controlled entirely by the emulator and possess lightweight autonomous control modules capable of executing planned trajectories and responding to surrounding traffic using the extracted features. Autonomous vehicles, by contrast, are controlled externally by real autonomous driving systems (e.g., Apollo). DTTF-Sim supports end-to-end integration with such systems, providing real-time sensor data (e.g., LiDAR, camera, radar, localization, and object ground truth) and receiving control commands (e.g., throttle, brake, steering). This setup enables closed-loop evaluation of perception, planning, and control modules under realistic and evolving traffic conditions.

The design and implementation of these modules are described in detail in Section 5.

## 5. System Design

### 5.1. Traffic Flow Feature Extractor Module

To ensure high-fidelity reproduction of real-world traffic dynamics, DTTF-Sim employs a dedicated traffic flow feature extractor module that systematically analyzes vehicle trajectories and decomposes them into three key categories: incoming features, longitudinal features, and lateral features. Each trajectory comprises a temporally ordered sequence of points, where each point includes timestamp, position (x,y), velocity, and lateral offset information. Algorithms 1 and 2 illustrate the process by which the traffic flow feature extractor derives traffic features from vehicle trajectory data.

**Figure 2:**
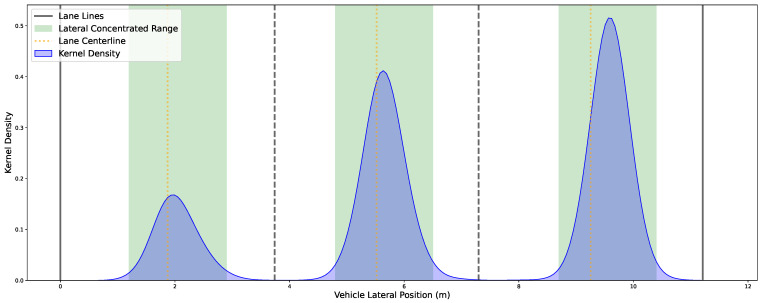
The kernel density of vehicle lateral position. The lateral position data of vehicles were obtained from the HighD dataset, specifically from the trajectories in locationId 3, which involved no lane-changing events. A total of 1559 trajectories were analyzed. The green zone in the figure represents the concentrated lateral driving region of vehicles, indicating their primary activity range within the lane. This region can be effectively used to define the start and end of lane-changing behaviors.

- Incoming features: describe the entry of vehicles into the observation zone during a fixed time interval *T*. Formally, for each interval [t,t+T], the extractor identifies all vehicles *i* with entry times $t_{i}\in \left[t,t+T\right]$ and records their initial speed $v_{i}$, geometric size $\left(l_{i},w_{i}\right)$, and lateral deviation $o_{i}$ from the lane centerline. These features determine the inflow characteristics of vehicles into the simulation environment.
- Longitudinal features: characterize lane-wise car-following behavior and include speed, gap, time headway, and acceleration. For all vehicles *i* in a given lane *L* during interval *T*, the extractor computes statistical moments (mean, standard deviation, max, min) of: speed $\left(v_{i}\right)$, gap $\left(g_{i}\right)$, time headway $\left(h_{i}=\frac{g_{i}}{v_{i}}\right)$, acceleration $\left(a_{i}\right)$. These features are used to directly parameterize a modified Krauss car-following model.
- Lateral Features: capture lane positioning and lane-changing behaviors. Lane-center deviation is defined as the vehicle’s lateral offset relative to the centerline of its current lane, as is shown in Figure 2. The extractor constructs a lateral kernel density map to determine the stable driving region for each lane, denoted $R_{\ell }=\left[y_{\mathrm{left}},y_{\mathrm{right}}\right]$. A lane-changing event is detected when a vehicle exits its current lane’s concentrated range and enters the target lane’s range. A complete lane-change trajectory is annotated with the following attributes: start and end times of the lane-change $\left(t_{s},t_{e}\right)$; original and target lanes $\left(\ell_{o},\ell_{t}\right)$; vehicle speed at the lane-change start $\left(v_{s}\right)$; and gaps to surrounding vehicles. These lateral features provide the foundation for modeling realistic lane-changing decisions and interactions among vehicles in adjacent lanes.

In summary, by extracting a structured set of traffic flow features from real-world trajectory data, the traffic flow feature extractor enables DTTF-Sim to construct dynamic, interactive, and statistically grounded traffic environments, thereby facilitating accurate digital twin mapping of real traffic flows.

Appendix A lists all the traffic flow features and their corresponding feature attributes used by DTTF-Sim.
**Algorithm 1:** Traffic Flow Longitudinal Feature Extraction Algorithm
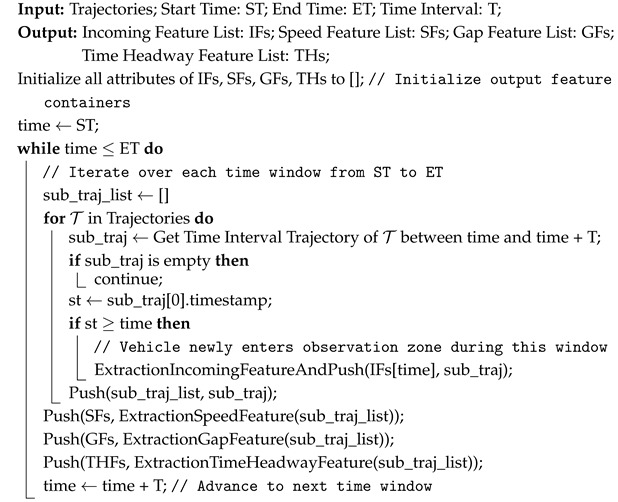


**Algorithm 2:** Traffic Flow Lane-Changing Feature Extraction Algorithm

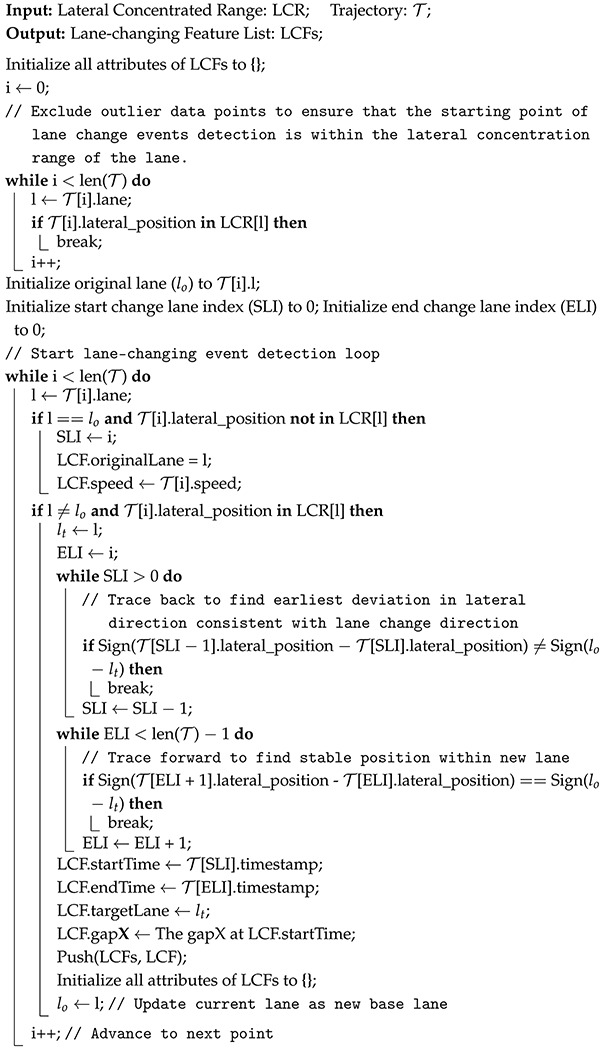



### 5.2. Traffic Flow Feature Emulator Module

DTTF-Sim divides the road into three functional zones: the flow generation zone, the observation zone, and the free zone, as shown in Figure 1. The emulator generates traffic background vehicles heading towards the observation zone based on vehicle incoming features in the flow generation flow generation zone. When vehicles enter the observation zone, background vehicles, under the guidance of the emulator, engage in dynamic interactions such as following behavior, lane changing, and collision avoidance control with surrounding vehicles to ensure the safety and stability of the traffic flow. To optimize computational resources, background vehicles are destroyed by the emulator when they enter the free zone. The lifecycle of a background vehicle begins in the flow generation zone, passes through the observation zone, and ends in the free zone.

**Figure 3:**
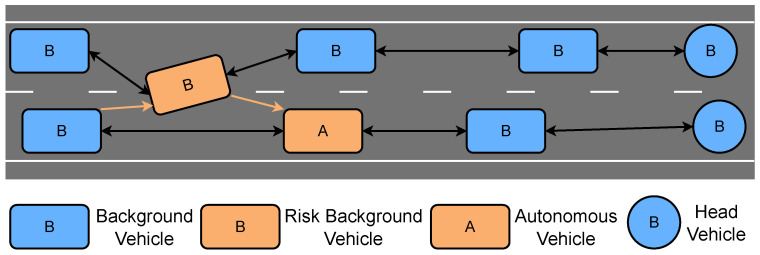
The lane model in DTTF-Sim. Each lane is encoded as a doubly linked list—circles are lead vehicles, squares followers. Black double arrows show safe predecessor--successor (car-following) relations, while orange nodes/arrows mark vehicles and pairs that require collision checks. To save computation, DTTF-Sim skips collision detection for vehicles in a direct following pair.

**Figure 4:**
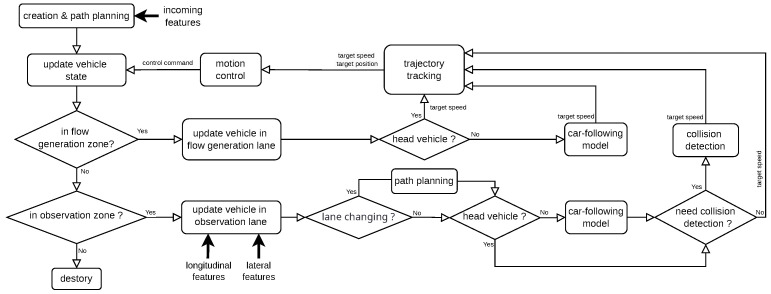
The lifecycle of background vehicle in DTTF-Sim.

In DTTF-Sim, each lane within a zone is modeled as a doubly linked list, as illustrated in Figure 3. To enhance computational efficiency, DTTF-Sim adopts an optimization strategy: collision detection is not performed between vehicles that are in a following relationship, as this relationship inherently ensures their safety. Background vehicles undergo a series of actions throughout their lifecycle, including creation, path planning, trajectory tracking, car-following behavior, lane changing, collision detection, lane centering correction, motion control and destruction, as is shown in Figure 4.

#### 5.2.1. Creation

The creation of background vehicles is driven by the incoming features. The incoming feature parameters include the geometric size (length, width), speed, and lane centerline offset of vehicles entering the observation zone. Based on these features, the emulator generates background vehicles in the simulation environment that closely resemble real vehicle behaviors and attributes, ensuring the accuracy and realism of the simulation.

#### 5.2.2. Path Planning

Path planning is performed during the creation and lane-change processes of background vehicles. In the creation phase, DTTF-Sim generates a smooth and continuous path for the background vehicle, extending from the flow generation zone to the observation zone. The path is then adjusted based on the lane centerline offset feature to ensure that the trajectory entering the observation zone aligns with real-world behavior. In the lane-change phase, the path planning module generates a guiding path for the background vehicle based on the lane-change time and speed, adhering to lane-change features. The lane centerline offset feature is applied again to ensure the similarity of the lane-change process. This design enables the background vehicle to perform efficient path planning and dynamic adjustments in complex traffic environments.

#### 5.2.3. Trajectory Tracking

The background vehicle always travels along the planned path, continuously adjusting its trajectory towards the next forward position point on the path. This process ensures that the behavior of vehicle in the simulation environment remains consistent with the planned path.

#### 5.2.4. Car-Following Behavior

The emulator drives the lane-following behavior within the current time interval based on the longitudinal features of the traffic flow. In DTTF-Sim, the car-following model extends the traditional krauss model [45,46]. While the parameters of the traditional krauss model is calibrated using dataset information, the parameters of the car-following model in DTTF-Sim (CFM-DTTF) are directly driven by the longitudinal features. The formula of CFM-DTTF is shown in Equation (1):

(1) $$\begin{array}{cc} v_{safe} & =v_{p}+\frac{g-v_{p}*{\bar{\phi }}_{min}}{\frac{v_{p}+v_{f}}{2*b}+{\bar{\phi }}_{min}}\\ v_{f} & =\min(v_{safe},v_{f}+{\bar{a}}_{max}\cdot \Delta t,{\bar{v}}_{max}) \end{array}$$

where ${\bar{\phi }}_{min}$ represents the real minimum value of the time headway feature; ${\bar{v}}_{max}$ represents the real max value of the speed feature; ${\bar{a}}_{max}$ represents the real max value of the acceleration feature; $v_{f}$ represents the vehicle speed; $v_{p}$ represents the speed of the preceding vehicle; *g* represents the gap between the vehicle and the preceding vehicle; *b* is the comfortable deceleration, $b=4\;m/s^{2}$;

#### 5.2.5. Lane-Changing

In real road environments, the reasons for a driver initiating a lane change are diverse, such as the desire to overtake for improved time efficiency, feeling fatigued and needing to switch to a slower lane, or adapting to changes in traffic flow [47]. To accurately reflect these real-world lane-changing behaviors, DTTF-Sim characterizes lane change events and details each event through the following attributes:

- startTime and endTime: The duration of the lane change event, from the start of the lane change to its completion.
- targetLane: The target lane direction, indicating which lane the vehicle will change to.
- speed and gapX: The aggressiveness of the lane change. speed represents the speed during the lane change, while gapX indicates the distance to the around vehicle. These two parameters together determine the urgency and safety of the lane change.

When the lane change feature is triggered, the reproducer selects the vehicle with the highest similarity to the actual lane change event from the original lane to perform the lane change operation, ensuring the accuracy of the simulated features. The similarity of lane change features is evaluated using the cosine similarity formula, which is provided by Equation (2). For a detailed explanation of these parameters, please refer to Appendix A.

(2) $$\begin{array}{cc} \bar{f} & =\left\{\bar{speed},\bar{gapX}\right\}\\ f & =\left\{speed,gapX\right\}\\ \mathrm{Similarity} & =\frac{\bar{f}*f}{\left|\right|\bar{f}\left|\right|\cdot \left||f|\right|} \end{array}$$

where $\bar{f}$ represents the real lane change feature; *f* denotes the lane change feature of the candidate vehicle in the simulation environment.

#### 5.2.6. Collision Detection

Twin vehicles identified by the emulator as risky vehicles or those affected by risky vehicles undergo collision detection. Specifically, autonomous vehicles and background vehicles performing lane changes are considered risky vehicles by the emulator. Additionally, the vehicles in front and behind the autonomous vehicle in the adjacent lanes, as well as the vehicles behind it in the same lane, are considered background vehicles affected by the autonomous vehicle. The vehicles in front and behind the target lane of a background vehicle changing lanes, as well as the vehicles behind it in its current lane, are considered background vehicles affected by the lane-changing vehicle. Since the remaining vehicles are in a safe following state, DTTF-Sim performs collision detection only for risky vehicles and those affected by them in order to save computational resources.

**Figure 5:**
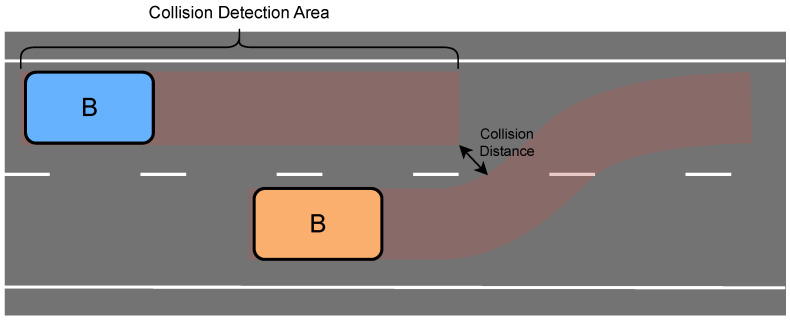
The collision detection schematic. In the illustration, orange vehicle indicates a vehicle with potential collision risk and blue vehicle indicates a vehicle with proceeding straight. The red area delineates the collision detection area. When performing a lane change, a potential collision-risk vehicle can interfere with a straight-going vehicle, as indicated by the overlap of their collision detection regions. DTTF-Sim evaluates the risk of collision by calculating the distance between these detection areas.

To enhance the timeliness of collision detection, DTTF-Sim employs the collision detection methodology illustrated in Figure 5. The red area delineates the collision detection area for background vehicles. This detection area extends rearward from the vehicle along its trajectory, with its length defined by Equation (3). DTTF-Sim assesses the risk of collision by calculating the minimum distance between two potential colliding vehicles.

(3) $$L_{\mathrm{CDA}}=l+a*v+b$$

where $L_{\mathrm{CDA}}$ represents the length of collision detection area; *l* represents the length of vehicle; *v* represents the speed of vehicle; *a* and *b* are constant numbers.

#### 5.2.7. Motion Control

The longitudinal PID controller generates throttle and brake commands to regulate the speed of background vehicles, while the lateral PID controller produces steering commands to control their direction. Together, these controllers ensure precise management of both the velocity and trajectory of the vehicles within the simulation environment.

#### 5.2.8. Destruction

When a twin vehicle exits the observation zone, the emulator destroys it to reduce unnecessary computational overhead.

### 5.3. Twin-Vehicle Module

**Figure 6:**
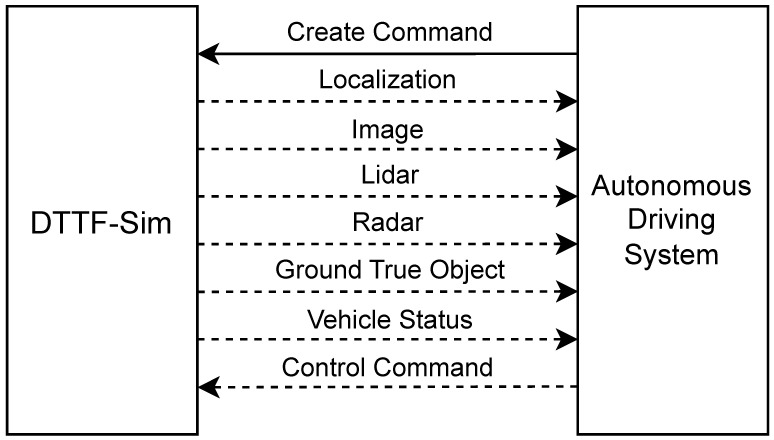
DTTF-Sim with autonomous driving system.

The twin vehicles are composed of an autonomous vehicle and a background vehicle. The construction method of background vehicles is detailed in the preceding section. Autonomous vehicles are controlled by an external autonomous driving system whose control logic is illustrated in Figure 6. DTTF-Sim provides users with comprehensive protocols for configuring autonomous vehicles and their sensors, allowing users to create them at any time. Once an autonomous vehicle is created, vehicle information and sensor data are transmitted to the autonomous driving system via network communication methods. This system generates control commands based on the received data to regulate the operation of vehicles within the simulation environment and facilitates interaction with other background vehicles.

To further clarify the system’s capabilities as summarized in Table 2, we provide the following explanation based on the design described in this section. DTTF-Sim supports long-term simulation through its feature-driven architecture, which extracts and sequentially reproduces real-world traffic characteristics over extended time intervals. Vehicle dynamics are considered by integrating a feature-driven car-following model and a lane-changing mechanism, which are parameterized using real trajectory data to maintain consistency with realistic kinematic behavior. Multi-vehicle interactions are modeled using a lane structure based on a doubly linked list, allowing background vehicles to perform coordinated actions such as lane-changing, collision checking, and car-following. The system also provides an end-to-end testing interface that enables external autonomous driving systems to receive sensor information and return control commands during simulation.

## 6. Implementation and Evaluations

**Figure 7:**
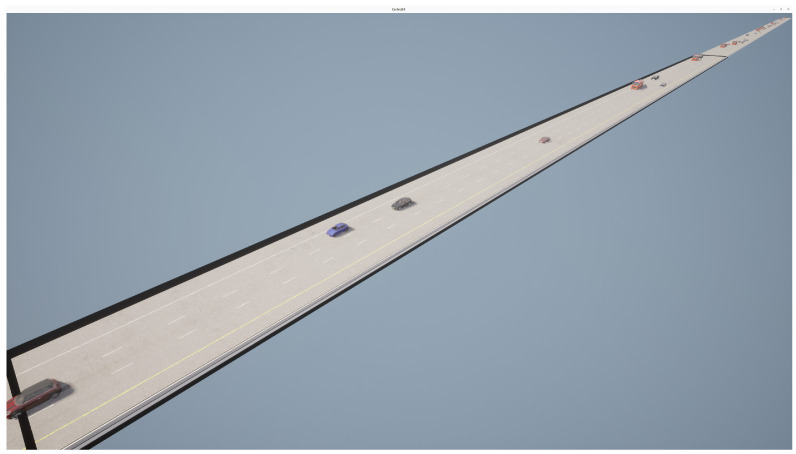
A digital twin environment in CARLA. This study employs the Carla engine to create a digital twin of the section with locationId 3 from the HighD dataset. The focus of this paper is on examining the performance of DTTF-Sim in simulating the dynamic features of traffic flow. Consequently, our work primarily centers on constructing the corresponding road network, without delving into the implementation of other more detailed static elements of the highway traffic environment. These additional environmental details fall outside the scope of this study. The video can be viewed at https://www.youtube.com/watch?v=7ycsj_db4H0 (accessed on 15 April 2025).

To evaluate the performance of DTTF-Sim in simulating real-world high-speed traffic flow, we constructed a simulation environment based on the data from a section of the HighD [48] dataset with a locationId of three, as shown in Figure 7. Please refer to the corresponding Table 3 for specific parameter settings.

**Figure 8:**
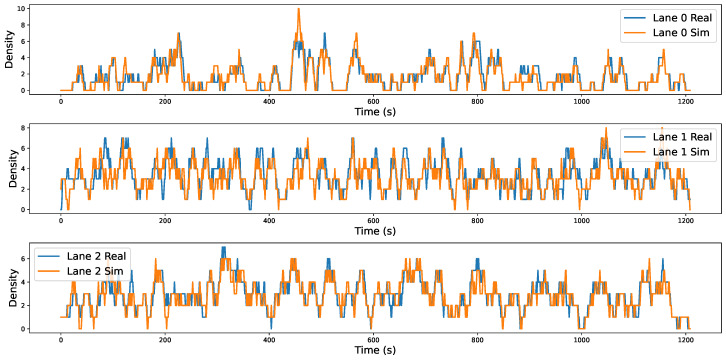
Lane density variation across lanes. In the simulation environment, there are three lanes, with Lane 0 being the innermost lane and Lane 2 the outermost lane. The lane density curve intuitively reflects the dynamic features of vehicles entering and leaving the lanes. We calculated the lane density in both the real-world and simulated environments at 1 s intervals. In the figure, the blue curve represents the lane density curve of the real-world environment, while the orange curve depicts the lane density curve of the simulated environment.

The variation in lane density reflects the dynamic behavior of vehicles within the observation area, including lane entries, exits, and lane-changing maneuvers. It thus serves as a key indicator for characterizing the evolution of traffic flow. As illustrated in Figure 8, the time-series curves of lane density provide an intuitive depiction of vehicle inflow and lane-changing behavior over time. In the figure, the blue curve represents lane density in the real-world environment, while the orange curve corresponds to data from the simulation environment. To quantitatively assess the accuracy of the simulation results, Table 4 presents the Mean Absolute Error (MAE) of lane density for each lane: 0.531 for Lane 1, 0.758 for Lane 2, and 0.509 for Lane 3. This metric evaluates the deviation between the simulated results and the real-world observations at each time step; smaller values indicate a closer match and suggest a higher level of consistency in reproducing dynamic traffic flow characteristics. The analysis shows that the simulated curves closely follow the trends of the real data, demonstrating that DTTF-Sim performs well in modeling vehicle inflow and lane-changing behaviors and is capable of reconstructing the interactive features of real-world traffic flow.

**Figure 9:**
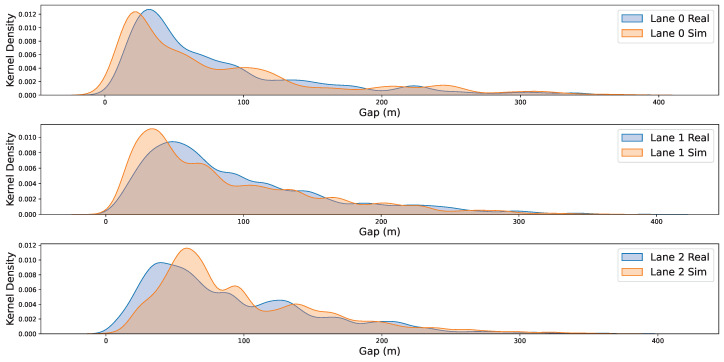
The kernel density curve of gap distribution. We created kernel density plots of the gap between vehicles for all three lanes, comparing both the real-world and simulation environments. Lane 0 represents the innermost lane, while Lane 2 is the outermost lane. In these plots, the blue curves correspond to the real-world environment, and the orange curves represent the simulation environment.

**Figure 10:**
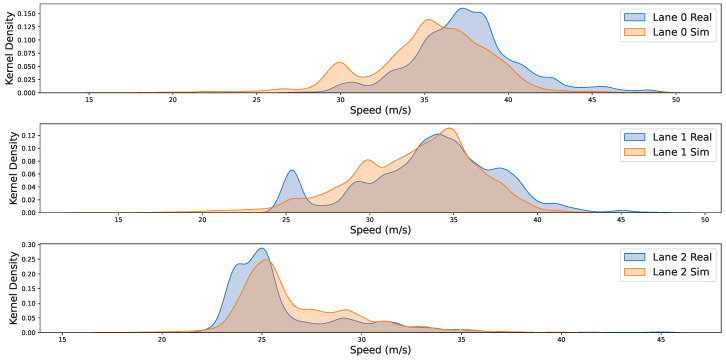
The kernel density curve of speed distribution. We created kernel density plots of the speed between vehicles for all three lanes, comparing both the real-world and simulation environments. Lane 0 represents the innermost lane, while Lane 2 is the outermost lane. In these plots, the blue curves correspond to the real-world environment, and the orange curves represent the simulation environment.

Furthermore, comparisons between the real-world and simulated environments concerning speed and gap distributions are shown in Figure 9 and Figure 10. The corresponding quantitative results are summarized in Table 5: the Kullback–Leibler (KL) divergence between real and simulated speed distributions is 0.252, 0.312, and 0.266 for Lanes 0–2, while the KL divergence for gap distributions is 0.225, 0.139, and 0.268, respectively. These uniformly small KL values corroborate the visual evidence, demonstrating that DTTF-Sim accurately reproduces the speed and gap characteristics of real traffic flow across all lanes.

The lane-changing events were executed with a **100%** success rate, achieving an average similarity of **0.868** to real-world features (where the maximum similarity is 1 and the minimum similarity is −1).

**Figure 11:**
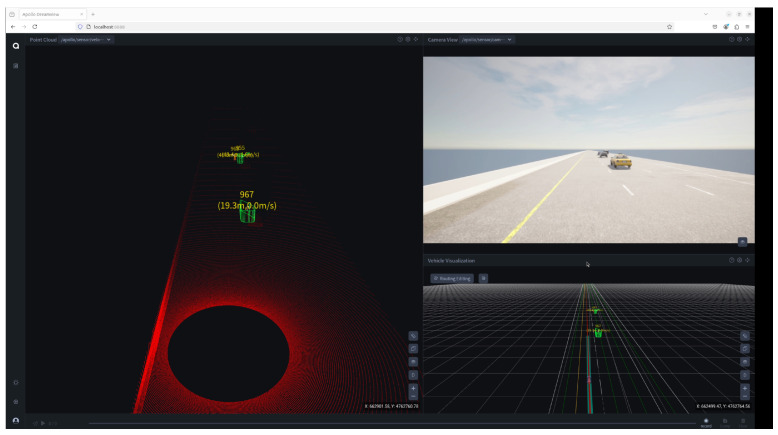
DTTF-Sim with Apollo. The video can be viewed at https://www.youtube.com/watch?v=zPRp-Gs7UI4 (accessed on 15 April 2025).

**Figure 12:**
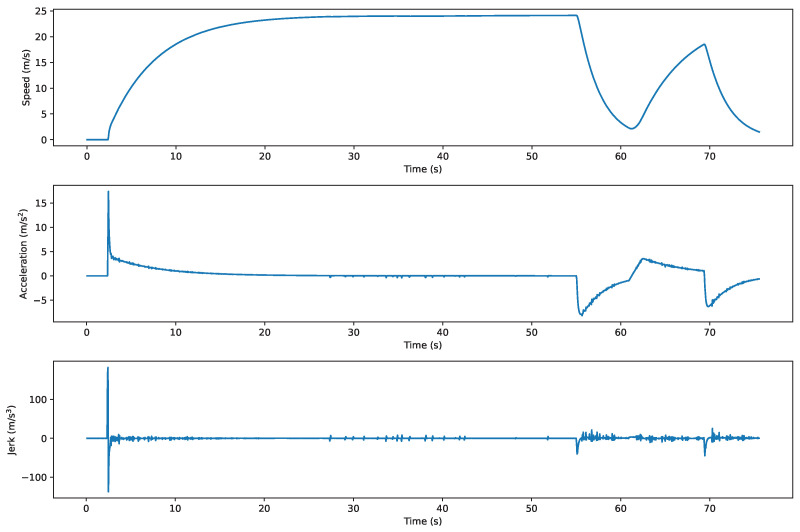
These curves illustrate the dynamic response of the simulated vehicle under Apollo’s control, showcasing real-time closed-loop interaction. Note that the abrupt spike in acceleration at the initial moment is a non-physical artifact caused by a sudden control signal in the CARLA environment.

Regarding the exploration of DTTF-Sim’s practicality, this study conducted a comprehensive end-to-end co-simulation analysis integrating DTTF-Sim with the Apollo autonomous driving system, as shown in Figure 11. In this process, DTTF-Sim is responsible for providing critical information to the Apollo autonomous driving system, including sensor data and vehicle chassis status, while Apollo generates corresponding vehicle control commands based on this information to drive the simulated vehicle operations. Figure 12 presents the velocity, acceleration, and jerk profiles of the simulated vehicle driven by control commands generated by the Apollo agent within DTTF-Sim. These results demonstrate that Apollo is capable of real-time interaction with DTTF-Sim, forming a closed-loop data system. Notably, this setup allows the autonomous driving system to effectively interact with simulated real traffic flow in a highly realistic environment, thereby verifying the feasibility and effectiveness of DTTF-Sim in practical applications. During the co-simulation, Apollo was able to receive real-time sensor data from DTTF-Sim, which transmitted sensor information at a frequency of 20 Hz. Apollo successfully generated smooth control trajectories. No abrupt braking or oscillatory lane-changing behavior was observed, indicating that its planning and control modules operated in a stable manner. The simulated background traffic created realistic interaction pressure, enabling the autonomous vehicle to perform adaptive maneuvers such as yielding and overtaking.

Furthermore, with respect to the “Real” dimension described in Table 2, the experimental results further support DTTF-Sim’s capability to reproduce key characteristics of real-world traffic. As shown in Figure 8, Figure 9 and Figure 10, the simulated lane density, speed distribution, and vehicle gap distribution closely follow the patterns observed in the HighD dataset. Quantitative metrics, including the Mean Absolute Error (MAE) and KL divergence, confirm the consistency between the simulated and actual data. Additionally, the average similarity of **0.868** in lane-changing behavior indicates that the feature-driven mechanisms in DTTF-Sim are capable of maintaining fidelity with real-world dynamics. These findings provide empirical support for the system’s design objective of realism in traffic behavior modeling.

The simulation experiments indicate that DTTF-Sim can accurately reproduce key characteristics of real-world traffic flow, including lane density, speed distribution, and inter-vehicle gap distribution. Quantitative metrics such as Mean Absolute Error (MAE) and Kullback–Leibler (KL) divergence confirm the simulator’s effectiveness in replicating dynamic traffic behaviors.

Since DTTF-Sim operates in a feature-driven manner, its simulation duration is inherently determined by the length of the injected traffic features. As a result, DTTF-Sim is capable of supporting long-term continuous simulations. In this study, the input dataset spans 20 min, and accordingly, DTTF-Sim provides continuous simulation over the same time period.

In addition, DTTF-Sim captures vehicle-level kinematic properties through a feature-guided car-following model and a similarity-based lane-changing mechanism. The system also supports full end-to-end closed-loop testing, as demonstrated by its integration with the Apollo autonomous driving system. In this setup, DTTF-Sim transmits real-time sensor and vehicle status data to Apollo, which then returns control commands, enabling interactive simulation within realistic background traffic.

It is worth noting, however, that the current implementation still has limitations. The supported road network is relatively simple and does not yet include complex highway structures such as ramps or intersections. Furthermore, behavior models are rule-based and do not yet incorporate human-like driving strategies powered by neural networks. These aspects will be addressed in future work to enhance the realism and generalizability of DTTF-Sim.

## 7. Conclusions

In this paper, we propose DTTF-Sim, an autonomous driving simulation system built on the concept of digital twins, designed to address key challenges in current autonomous driving system testing—such as high costs, significant risks, and limited scalability. Built on the CARLA simulation platform and driven by real-world traffic data, DTTF-Sim supports realistic traffic flow simulation, long-duration testing, multi-vehicle interaction, and end-to-end closed-loop evaluation. We validate its effectiveness in replicating real-world traffic behaviors using the HighD dataset and further demonstrated its applicability through integration with the Apollo autonomous driving system.

Despite achieving the core simulation functionalities, DTTF-Sim still has several limitations. First, the current supported road network structure is relatively simple and does not yet include complex highway elements such as ramps, merges, or intersections that are commonly encountered in real-world scenarios. Second, the car-following and lane-changing behaviors are still based on rule-based models, lacking human-like behavior modeling using neural networks. Additionally, the experimental evaluation lacks sufficient critical analysis of the simulator’s performance. In the future, we plan to incorporate a broader set of representative scenarios and failure case analyses to enhance the comprehensiveness and reliability of the system evaluation.

Looking ahead, we will continue to improve DTTF-Sim by expanding the supported road network structures and driving behavior models, with a particular focus on integrating high-fidelity, human-like driving strategies based on deep learning. We also aim to improve compatibility with mainstream autonomous driving systems. Furthermore, we plan to open access to the platform and ensure compatibility with a wider range of traffic datasets, enabling researchers to simulate more diverse and realistic traffic scenarios and contributing to the advancement of autonomous driving testing technologies.

## Figures and Tables

**Table 1:** Comparison of Different Types of Autonomous Driving Simulators.

Simulator Type	Advantages	Limitations	Typical Simulators
Flow-based	Support large-scale traffic modeling and traffic management evaluation; Good scalability and efficiency.	Simplified vehicle models as point-mass; Cannot simulate realistic vehicle dynamics and interaction.	SUMO, VISSIM, Aimsun, Paramics
Scenario-based	Support dynamic scene rendering and vehicle kinematics; Suitable for perception, planning, and control testing.	Depend on predefined scripts and behavior trees; Limited diversity and spontaneity in interaction.	CARLA, VTD, LGSVL, NVIDIA Drive
Vehicle Dynamics	Accurate modeling of vehicle control systems and dynamics; Ideal for component- level validation.	Lack multi-vehicle interaction modeling and real-world traffic scenes; Not suitable for system- level evaluation.	CarSim, Simulink, dSPACE, CarMaker
Data-driven	Leverage real-world data to learn complex driving behaviors and generate diverse scenarios. Capable of producing traffic scenarios with high realism.	Vehicles modeled as point masses, lacking kinematic fidelity; Weak support for sensor-level simulation; limited to planning- level evaluation.	SimNet, TrafficGen, nuPlan, Waymo
DTTF-Sim	Supports long-term simulation with digital twin mapping; Preserves vehicle dynamics; enables closed-loop integration with ADS.	Currently lacks complex road network support (e.g., ramps, intersections); Behavior models are rule-based.	-

**Table 2:** Comparisons with Existing Simulators.

Simulator	Long Term	Vehicle Dynamic	Diverse Interaction	End-to-End	Real
Flow-based	+	-	∘	-	∘
Scenario-based	∘	+	∘	∘	∘
Vehicle-based	∘	+	-	-	∘
Data-based	∘	∘	+	-	+
DTTF-Sim	+	+	∘	+	+

+ Good Support; ∘ Basic Support; - No Support

**Table 3:** Experiment Parameters.

Parameter	Value
CPU	i7-12700KF
GPU	NVIDIA RTX 3080 Ti
Time Interval	1 s
Total Time	20-min
Vehicle Count	1211
Lane-changing Count	99
Lane Number	3
The Length of Flow Generation Zone	1000 m
The Length of Observation Zone	400 m

**Table 4:** Mean Absolute Error (MAE) for Lane Density.

Lane	0	1	2
MAE	0.531	0.758	0.509

**Table 5:** KL Divergence for Lane Speed and Gap Distribution.

Lane	0	1	2
Speed	0.252	0.312	0.266
Gap	0.225	0.139	0.268

## Data Availability

The research data supporting this publication are available from the corresponding author upon reasonable request.

## Appendix A

This appendix provides an overview of the attributes of traffic flow features in DTTF-Sim, along with their corresponding numerical types and meanings.

- Incoming Feature:

- speed [double]: The speed of the vehicle as it enters the observation zone.
- size [double, double]: The size of the vehicle, including length and width.
- offset [double]: The average offset of the vehicle from the lane center line within the observation zone.

- Speed Feature:

- max [double]: The maximum speed of vehicles traveling on lane L within the time interval *T*.
- min [double]: The minimum speed of vehicles traveling on lane L within the time interval *T*.
- mean [double]: The mean speed of vehicles traveling on lane L within the time interval *T*.
- std [double]: The standard deviation of vehicle speeds on lane L within the time interval *T*.

- Gap Feature:

- max [double]: The maximum gap of vehicles traveling on lane L within the time interval *T*.
- min [double]: The minimum gap of vehicles traveling on lane L within the time interval *T*.
- mean [double]: The mean gap of vehicles traveling on lane L within the time interval *T*.
- std [double]: The standard deviation of gap on lane L within the time interval *T*.

- Time Headway Feature:

- max [double]: The maximum time headway of vehicles traveling on lane L within the time interval *T*.
- min [double]: The minimum time headway of vehicles traveling on lane L within the time interval *T*.
- mean [double]: The mean time headway of vehicles traveling on lane L within the time interval *T*.
- std [double]: The standard deviation of time headway on lane L within the time interval *T*.

- Acceleration Feature:

- max [double]: The maximum accelerattion of vehicles traveling on lane L within the time interval *T*.
- min [double]: The minimum accelerattion of vehicles traveling on lane L within the time interval *T*.
- mean [double]: The mean accelerattion of vehicles traveling on lane L within the time interval *T*.
- std [double]: The standard deviation of accelerattion on lane L within the time interval *T*.

- Lane-changing Feature:

- startTime [int]: The start time of the lane change event.
- endTime [int]: The end time of the lane change event.
- originalLane [int]: The original lane time of the lane change event.
- targetLane [int]: The target lane time of the lane change event.
- speed [double]: The speed when vehicle start lane-changing.
- gapOFollowing [double]: The gap between the lane-changing vehicle and the following vehicle in the original lane.
- gapOPreceding [double]: The gap between the lane-changing vehicle and the preceding vehicle in the original lane.
- gapTFollowing [double]: The gap between the lane-changing vehicle and the following vehicle in the target lane.
- gapTPreceding [double]: The gap between the lane-changing vehicle and the preceding vehicle in the target lane.

- Lateral Concentrated Range:

- left [double]: The left boundary of the Lateral Concentrated Range.
- right [double]: The right boundary of the Lateral Concentrated Range

## References

[1] Kalra N., Paddock S.M. (2016). Driving to Safety: How Many Miles of Driving Would It Take to Demonstrate Autonomous Vehicle Reliability?. Transp. Res. Part A Policy Pract..

[2] Alvarez Lopez P., Banse A., Barthauer M., Behrisch M., Couéraud B., Erdmann J., Flötteröd Y.-P., Hilbrich R., Nippold R., Wagner P. (2024). Simulation of Urban Mobility (SUMO).

[3] Fellendorf M., Vortisch P., Barceló J. (2010). Microscopic Traffic Flow Simulator VISSIM. Fundamentals of Traffic Simulation.

[4] Casas J., Ferrer J.L., Garcia D., Perarnau J., Torday A., Barceló J. (2010). Traffic Simulation with Aimsun. Fundamentals of Traffic Simulation.

[5] Cameron G.D.B., Duncan G.I.D. (1996). PARAMICS—Parallel Microscopic Simulation of Road Traffic. J. Supercomput..

[6] von Neumann-Cosel K., Dupuis M., Weiss C. Virtual Test Drive—Provision of a Consistent Tool-Set for [D,H,S,V]-in-the-Loop. Proceedings of the DSC 2009 Europe.

[7] Dosovitskiy A., Ros G., Codevilla F., Lopez A., Koltun V. CARLA: An Open Urban Driving Simulator. Proceedings of the 1st Annual Conference on Robot Learning.

[8] Chen H., Ren H., Li R., Yang G., Ma S. (2022). Generating Autonomous Driving Test Scenarios Based on OpenSCENARIO. Proceedings of the 2022 9th International Conference on Dependable Systems and Their Applications (DSA).

[9] Bergamini L., Ye Y., Scheel O., Chen L., Hu C., Del Pero L., Osinski B., Grimmett H., Ondruska P. (2021). SimNet: Learning Reactive Self-Driving Simulations from Real-World Observations. arXiv.

[10] Feng L., Li Q., Peng Z., Tan S., Zhou B. (2022). TrafficGen: Learning to Generate Diverse and Realistic Traffic Scenarios. arXiv.

[11] Nuli S., Mathew T.V. (2013). Online Coordination of Signals for Heterogeneous Traffic Using Stop Line Detection. Procedia—Soc. Behav. Sci..

[12] Wang J., Chai R., Wu Q. (2014). Changing Lane Probability Estimating Model Based on Neural Network. Proceedings of the 26th Chinese Control and Decision Conference (2014 CCDC).

[13] Chen C., Cao Y., Tang K., Li K. (2021). Dynamic Path Flow Estimation Using Automatic Vehicle Identification and Probe Vehicle Trajectory Data: A 3D Convolutional Neural Network Model. J. Adv. Transp..

[14] Li D., Hou Z. (2021). Perimeter Control of Urban Traffic Networks Based on Model-Free Adaptive Control. IEEE Trans. Intell. Transport. Syst..

[15] Farah H., Postigo I., Reddy N., Dong Y., Rydergren C., Raju N., Olstam J. (2023). Modeling Automated Driving in Microscopic Traffic Simulations for Traffic Performance Evaluations: Aspects to Consider and State of the Practice. IEEE Trans. Intell. Transport. Syst..

[16] Abbaszadeh Shahri A., Pashamohammadi F., Asheghi R., Abbaszadeh Shahri H. (2022). Automated Intelligent Hybrid Computing Schemes to Predict Blasting Induced Ground Vibration. Eng. Comput..

[17] Gao L., Bai W., Leary R., Varadarajan K., Brennan S. (2021). ROS Integration of External Vehicle Motion Simulations with an AIMSUN Traffic Simulator as a Tool to Assess CAV Impacts on Traffic. IFAC-PapersOnLine.

[18] Sykes P., Barceló J. (2010). Traffic Simulation with Paramics. Fundamentals of Traffic Simulation.

[19] Cortés C.E., Stefoni B. (2023). Trajectory Simulation of Emergency Vehicles and Interactions with Surrounding Traffic. J. Adv. Transp..

[20] Luo G., Shao C., Cheng N., Zhou H., Zhang H., Yuan Q., Li J. (2024). EdgeCooper: Network-Aware Cooperative LiDAR Perception for Enhanced Vehicular Awareness. IEEE J. Select. Areas Commun..

[21] Tang X., Zhong G., Li S., Yang K., Shu K., Cao D., Lin X. (2023). Uncertainty-Aware Decision-Making for Autonomous Driving at Uncontrolled Intersections. IEEE Trans. Intell. Transport. Syst..

[22] Hou N., Ding N., Zhang H., Ran C., Wu H. (2023). Comparative Safety and Stability Assessment of Autonomous Vehicle Lane-Changing Strategies in Mixed Traffic at Urban Expressway Weaving Section. Proceedings of the 2023 IEEE 26th International Conference on Intelligent Transportation Systems (ITSC).

[23] Xiao T., Chen S., Zhu K., Huang R., Li A., Zheng N., Xin J. (2022). Injection Simulation: An Efficient Validation Framework for Autonomous Driving System. Proceedings of the 2022 IEEE 25th International Conference on Intelligent Transportation Systems (ITSC).

[24] Masi S., Xu P., Bonnifait P. (2022). Roundabout Crossing With Interval Occupancy and Virtual Instances of Road Users. IEEE Trans. Intell. Transport. Syst..

[25] Kirchengast M., Watzenig D. (2024). A Depth-Buffer-Based Lidar Model With Surface Normal Estimation. IEEE Trans. Intell. Transport. Syst..

[26] Muckenhuber S., Holzer H., Rubsam J., Stettinger G. (2019). Object-Based Sensor Model for Virtual Testing of ADAS/AD Functions. Proceedings of the 2019 IEEE International Conference on Connected Vehicles and Expo (ICCVE).

[27] Zhu Y., Wang J., Guo X., Meng F., Liu T. (2023). Functional Testing Scenario Library Generation Framework for Connected and Automated Vehicles. IEEE Trans. Intell. Transport. Syst..

[28] Kaljavesi G., Kerbl T., Betz T., Mitkovskii K., Diermeyer F. (2024). CARLA-Autoware-Bridge: Facilitating Autonomous Driving Research with a Unified Framework for Simulation and Module Development. Proceedings of the 2024 IEEE Intelligent Vehicles Symposium (IV).

[29] Luan W., Ding Q., Wu Y. (2023). Research on Integrated Environment of Autonomous Vehicle Simulation Based on Apollo. Proceedings of the 2023 5th International Conference on Robotics, Intelligent Control and Artificial Intelligence (RICAI).

[30] Zhou Z., Rother C., Chen J. (2023). Event-Triggered Model Predictive Control for Autonomous Vehicle Path Tracking: Validation Using CARLA Simulator. IEEE Trans. Intell. Veh..

[31] Li D., Okhrin O. (2023). Modified DDPG Car-Following Model with a Real-World Human Driving Experience with CARLA Simulator. Transp. Res. Part C Emerg. Technol..

[32] Ramakrishna S., Luo B., Kuhn C.B., Karsai G., Dubey A. (2022). ANTI-CARLA: An Adversarial Testing Framework for Autonomous Vehicles in CARLA. Proceedings of the 2022 IEEE 25th International Conference on Intelligent Transportation Systems (ITSC).

[33] Bu T., Zhang X., Mertz C., Dolan J.M. (2021). CARLA Simulated Data for Rare Road Object Detection. Proceedings of the 2021 IEEE International Intelligent Transportation Systems Conference (ITSC).

[34] Wen L., Fu D., Mao S., Cai P., Dou M., Li Y., Qiao Y. (2023). LimSim: A Long-Term Interactive Multi-Scenario Traffic Simulator. arXiv.

[35] Jansen W., Verreycken E., Schenck A., Blanquart J.-E., Verhulst C., Huebel N., Steckel J. (2023). Cosys-AirSim: A Real-Time Simulation Framework Expanded for Complex Industrial Applications. arXiv.

[36] Duan J., Wang Y., Ding J., Deng W. (2023). Digital Twin Test Method for Autonomous Vehicles Based on PanoSim.

[37] Ying L., Long J., Shang M., Wang X., Wang F.-Y. (2024). ACP-Incorporated Perturbation-Resistant Neural Dynamics Controller for Autonomous Vehicles. IEEE Trans. Intell. Veh..

[38] Liang J., Tian Q., Feng J., Pi D., Yin G. (2024). A Polytopic Model-Based Robust Predictive Control Scheme for Path Tracking of Autonomous Vehicles. IEEE Trans. Intell. Veh..

[39] Huang Y., Ding H., Zhang Y., Wang H., Cao D., Xu N., Hu C. (2020). A Motion Planning and Tracking Framework for Autonomous Vehicles Based on Artificial Potential Field Elaborated Resistance Network Approach. IEEE Trans. Ind. Electron..

[40] Quirynen R., Safaoui S., Di Cairano S. (2025). Real-Time Mixed-Integer Quadratic Programming for Vehicle Decision-Making and Motion Planning. IEEE Trans. Contr. Syst. Technol..

[41] Dutta T., Reddy D.S., Rajalakshmi P. (2024). Real-Time Deep Learning Based Safe Autonomous Navigation. Proceedings of the 2024 8th International Conference on Robotics, Control and Automation (ICRCA).

[42] Caesar H., Kabzan J., Tan K.S., Fong W.K., Wolff E., Lang A., Fletcher L., Beijbom O., Omari S. (2022). NuPlan: A Closed-Loop ML-Based Planning Benchmark for Autonomous Vehicles. arXiv.

[43] Montali N., Lambert J., Mougin P., Kuefler A., Rhinehart N., Li M., Gulino C., Emrich T., Yang Z., Whiteson S. (2023). The Waymo Open Sim Agents Challenge. arXiv.

[44] Tao F., Liu W., Zhang M., Hu T., Luo Y. (2019). Five-Dimension Digital Twin Model and Its Ten Applications. Comput. Integr. Manuf. Syst..

[45] Krauss S. (1998). Microscopic Modeling of Traffic Flow: Investigation of Collision Free Vehicle Dynamics. Ph.D. Thesis.

[46] Krauss S., Wagner P., Gawron C. (1997). Metastable States in a Microscopic Model of Traffic Flow. Phys. Rev. E.

[47] Gipps P.G. (1986). A Model for the Structure of Lane-Changing Decisions. Transp. Res. Part B Methodol..

[48] Krajewski R., Bock J., Kloeker L., Eckstein L. (2018). The highD Dataset: A Drone Dataset of Naturalistic Vehicle Trajectories on German Highways for Validation of Highly Automated Driving Systems. Proceedings of the 2018 21st International Conference on Intelligent Transportation Systems (ITSC).

